# Exploring Research Hotspots and Emerging Trends in Mitochondria and Chronic Pain: A Bibliometric and Visualized Analysis From 2004 to 2024

**DOI:** 10.1155/prm/6810455

**Published:** 2025-10-01

**Authors:** Qian Wang, Tao Wu, Ming Hu, Yuxin Liu, Feng Jiang, Yimeng Xia

**Affiliations:** ^1^Department of Pediatrics, Shanghai General Hospital, Shanghai Jiao Tong University School of Medicine, Shanghai, China; ^2^Pediatric Department, Starkids Children's Hospital, New Hong Qiao Campus for Children's Hospital of Fudan University, Shanghai, China; ^3^Department of Anesthesiology, Ruijin Hospital, Shanghai Jiaotong University School of Medicine, Shanghai, China; ^4^Diagnostics and Therapeutics of Intractable Diseases, Intractable Disease Research Center, Graduate School of Medicine, Juntendo University, Tokyo, Japan; ^5^Department of Neonatology, Shanghai Key Laboratory of Reproduction and Development, Shanghai Key Laboratory of Female Reproductive Endocrine Related Diseases, Obstetrics & Gynecology Hospital of Fudan University, Shanghai, China

**Keywords:** bibliometric analysis, citation analysis, mitochondria, pain, research trends

## Abstract

**Background:**

Mitochondria play a crucial role in various cellular processes, and their dysfunction has been increasingly recognized as a significant factor in the pathophysiology of chronic pain. Despite growing interest in this area, a comprehensive bibliometric analysis of the research trends, key themes, and influential contributions in the study of mitochondria and pain has not been conducted. This study aims to fill this gap by providing a detailed overview of the research landscape from 2004 to 2024.

**Methods:**

A comprehensive literature search was performed using the Web of Science Core Collection database to identify relevant studies published between January 1, 2004, and June 24, 2024. A total of 1995 articles were identified and included in the analysis. Bibliometric tools, including CiteSpace, VOSviewer, and RStudio, were employed to analyze and visualize data related to cocitation networks, keyword co-occurrence, and clustering patterns. The analysis focused on identifying research trends, key authors, influential journals, and emerging themes within the field.

**Results:**

The analysis revealed a significant increase in research activity on mitochondria and pain, particularly after 2011, with the number of publications rising from 74 in 2015 to 171 in 2020, representing a 131% growth during this period. Key themes identified include mitochondrial dysfunction, oxidative stress, neuroinflammation, autophagy, and mitochondrial biogenesis. The United States, China, Germany, and the United Kingdom emerged as leading contributors to the field, with prominent institutions such as Harvard University and the University of California playing central roles in collaborative networks. Influential authors and foundational studies were identified through cocitation analysis, highlighting the interdisciplinary nature of the research.

**Conclusion:**

This bibliometric analysis provides a comprehensive overview of the evolving research landscape in mitochondria and pain, highlighting key trends, influential contributions, and emerging research directions. The findings underscore the growing recognition of mitochondrial dysfunction as a critical factor in pain mechanisms and suggest that future research should focus on translating basic findings into clinical practice while expanding global collaborations.

## 1. Introduction

Pain is a complex and subjective sensory and emotional experience that involves multiple components, including nociceptive, emotional, cognitive, and social factors [1, 2]. Unlike acute pain, which serves as a protective mechanism signaling potential tissue damage, chronic pain persists beyond the usual course of healing and can significantly impair quality of life. Chronic pain encompasses various types, such as visceral, inflammatory, neuropathic, and headache pain [3, 4]. Emerging evidence indicates that mitochondrial dysfunction through mechanisms such as energy deficits, oxidative stress, and proinflammatory signaling may contribute to the pathogenesis of these diverse pain types, highlighting mitochondria as a potential common therapeutic target. Visceral pain, often associated with conditions like Crohn's disease, is a significant clinical concern due to its complex nature and the limited effectiveness of current interventions. Studies suggest that mitochondrial dysfunction in visceral sensory neurons can disrupt energy metabolism and enhance oxidative stress, potentially contributing to visceral hypersensitivity and chronic symptoms [5]. Neuropathic pain, exemplified by distal symmetric polyneuropathy in diabetes, profoundly affects patient quality of life, with treatments offering only partial relief. Research has shown that impaired mitochondrial dynamics, excessive reactive oxygen species (ROS) production, and mitochondrial DNA damage in dorsal root ganglion neurons are closely linked to neuronal injury and increased pain sensitivity in neuropathic conditions [6]. Inflammatory pain arises from tissue inflammation and is commonly seen in disorders such as rheumatoid arthritis [7, 8]. Mitochondria are actively involved in inflammatory signaling, with mitochondrial-derived ROS and mitochondrial DNA acting as proinflammatory mediators that can sustain nociceptor sensitization and prolong inflammatory pain [5]. Headaches, including migraines, are prevalent and debilitating, with underlying mechanisms still being investigated [9]. Each pain type presents distinct therapeutic challenges, underscoring the need for more effective and tailored treatment strategies.

Mitochondria are essential organelles responsible for producing over 90% of cellular energy through oxidative phosphorylation. They play vital roles in various cellular processes, including ATP production, biosynthetic pathways, redox homeostasis, ion homeostasis, oxygen sensing, signaling, and the regulation of programmed cell death [10, 11]. Since their discovery by Altmann in 1894, mitochondrial dysfunction has been implicated in numerous neurological disorders affecting both the central and peripheral nervous systems. The five major mitochondrial functions: energy generation, ROS production, mitochondrial permeability transition pore (MPTP) regulation, apoptotic pathways, and intracellular calcium mobilization are critical in understanding the pathogenesis of chronic pain [12, 13].

Mitochondria are critical in the pathogenesis of chronic pain through multiple mechanisms. Their dysfunction can impair ATP production, leading to disruptions in cellular energy homeostasis that contribute to both neuropathic and inflammatory pain. Additionally, mitochondria can produce excessive ROS, a group of highly reactive molecules derived from oxygen metabolism. Overproduction of ROS leads to oxidative stress, which sensitizes nociceptors and enhances pain perception [6, 14]. The regulation of intracellular calcium levels by mitochondria is also crucial, as calcium dysregulation can increase neuronal excitability and pain sensitivity. Furthermore, the MPTP has been implicated in apoptosis, a process associated with neuropathic pain. Experimental studies have shown that pharmacological inhibition of MPTP, for example, with cyclosporine A, may attenuate pain behaviors in preclinical models [15, 16]. Overall, these mechanisms highlight the potential of targeting mitochondrial function as a therapeutic strategy for managing chronic pain.

Bibliometrics is the quantitative analysis of scientific literature, providing insights into research trends, impact, and the development of specific fields over time. This method involves the statistical evaluation of articles, citations, and other academic outputs to identify patterns and research hotspots. Bibliometric analysis helps researchers understand the evolution of scientific domains, assess the influence of particular studies or researchers, and explore collaborations and network structures within the scientific community [17]. Compared with traditional narrative reviews, bibliometric analysis offers an objective and systematic approach, reducing selection bias and allowing for reproducible mapping of the research landscape. It can reveal how a field has evolved over time, uncover collaborative networks among countries, institutions, and authors, and highlight emerging topics that may shape future investigations. We chose this method because the literature on mitochondria and pain is extensive, multidisciplinary, and rapidly expanding, making it difficult for conventional reviews to comprehensively capture its development. A bibliometric approach enables us to quantitatively summarize two decades of research, identify the most influential works and contributors, and provide evidence-based insights into research gaps and future directions. Therefore, this study aims to fill that gap by providing a systematic bibliometric and visualized analysis of the global research trends on mitochondria and chronic pain from 2004 to 2024.

## 2. Materials and Methods

### 2.1. Data Sources and Search Strategy

**Figure 1:**
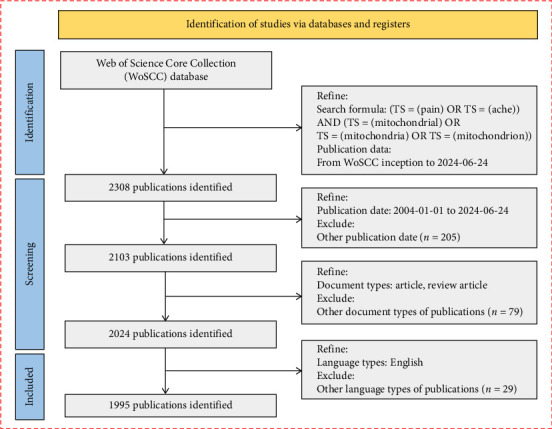
Flowchart of the study selection process. This figure outlines the steps taken to identify, screen, and include studies in the bibliometric analysis, based on specific inclusion and exclusion criteria.

A comprehensive literature search was conducted using the Web of Science Core Collection database to identify relevant studies on the relationship between mitochondria and pain. The search formula used was: (TS = (pain) OR TS = (ache)) AND (TS = (mitochondrial) OR TS = (mitochondria) OR TS = (mitochondrion)). The search was restricted to publications dated from January 1, 2004, to June 24, 2024. We focused on selecting articles that were categorized as original research or review articles to ensure a comprehensive overview of the existing literature. Only articles published in English were included to maintain consistency and ensure that the content was accessible. The flowchart of the study selection process is shown in Figure 1.

### 2.2. Data Analysis and Visualization

We analyzed publications from January 1, 2004, to June 24, 2024. The starting point of 2004 was selected to capture the modern phase of mitochondria-related pain research, coinciding with the wider adoption of high-throughput molecular techniques and the availability of more comprehensive bibliometric records in the Web of Science Core Collection. The end date was set to include the most recent complete dataset available at the time of analysis, ensuring that current research trends were adequately represented.

Data were analyzed and visualized using CiteSpace 6.2.R4. CiteSpace is a widely used bibliometric visualization software designed to identify emerging trends and pivotal points in scientific literature. It supports multiple functions, including cocitation analysis (measuring the frequency with which two documents are cited together, thereby revealing relationships between topics), cluster analysis (grouping similar items such as references or keywords into thematic clusters), and burst detection (identifying references or keywords that receive a sudden increase in attention).

## 3. Results

### 3.1. Trends in Mitochondria and Pain-Related Publications

**Figure 2:**
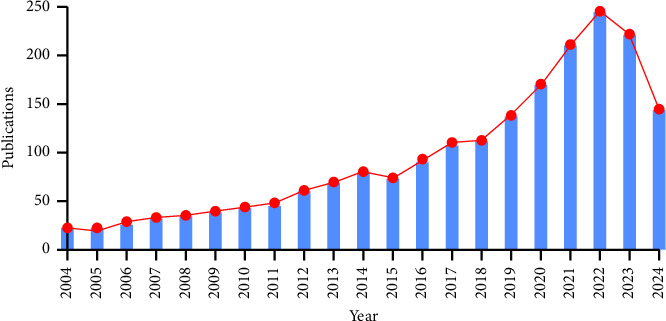
Trends in mitochondria and pain-related publications from 2004 to 2024. The figure shows the annual number of publications over time, highlighting significant increases in research activity, particularly after 2015.

Our bibliometric analysis revealed a clear upward trend in publications focusing on the relationship between mitochondria and pain from 2004 to 2024. The early years (2004–2010) showed minimal research activity, but a significant increase began around 2011.  Figure 2 highlighted a marked surge in publications between 2015 and 2020, indicating a growing recognition of the role of mitochondrial dysfunction in pain mechanisms. The trend line continued to rise through 2024, emphasizing that this area remained a prominent research focus, likely driven by advancements in mitochondrial science and the ongoing search for novel pain management strategies.

### 3.2. International and Institutional Collaboration in Mitochondria and Pain Research

**Figure 3:**
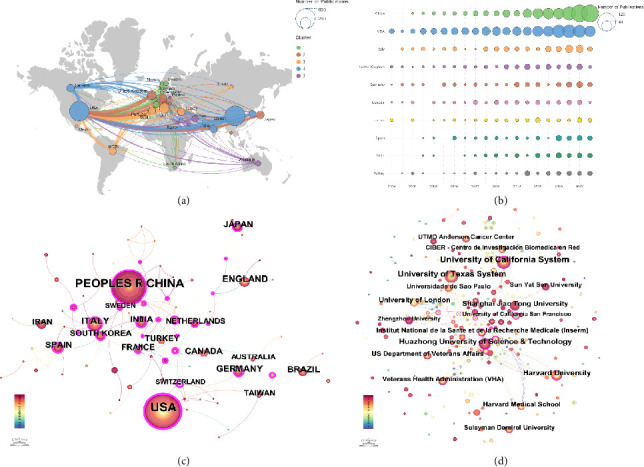
International and institutional collaboration in mitochondria and pain research. (a) Global map of international collaborations, showing the network of research partnerships across countries/regions. (b) Top 10 countries/regions by publication volume, illustrating the leading contributors to the research field. (c) Co-occurrence network of countries/regions in joint research efforts, highlighting central nodes like the United States, China, and the United Kingdom. (d) Institutional co-occurrence network, showcasing collaborations among leading research institutions worldwide.

Figure 3 provides a detailed analysis of international and institutional collaborations in mitochondria and pain research from 2004 to 2024. Figure 3(a) displays a global map of international collaborations, highlighting the extensive network of partnerships across various countries/regions. The map showed that the United States, China, and specific European nations like Germany and the United Kingdom were key players in this research field, frequently engaging in collaborative efforts. Figure 3(b) presents the top 10 countries by publication volume, the United States ranked first in publication volume with 541 articles (27.1%), followed closely by China with 510 articles (25.5%), reflecting their central roles in this research field. This data underscored the significant contributions of these countries/regions to the global research landscape on mitochondria and pain. Figure 3(c) illustrates the co-occurrence of countries/regions in joint research efforts. The United States, China, and the United Kingdom appeared as central nodes in this network, indicating their pivotal role in fostering international collaborations that drove research progress in this area. Figure 3(d) focuses on institutional co-occurrence, revealing collaborations between leading research institutions worldwide. Notable institutions such as Harvard University, the University of California, and the University of Texas were prominent in the network, demonstrating strong collaborative ties.  Table 1 presents the top 10 countries/regions and institutions on research of mitochondria and pain.

### 3.3. Author Collaboration and Co-Citation Analysis in Mitochondria and Pain Research

**Figure 4:**
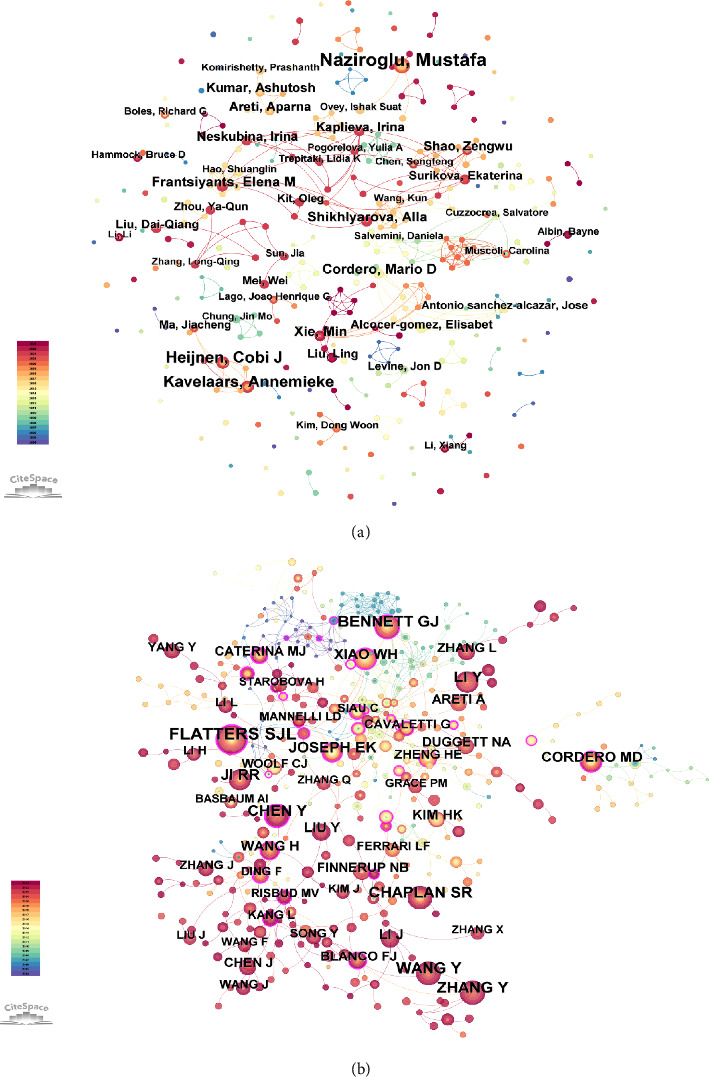
Author collaboration and cocitation analysis in mitochondria and pain research. (a) Coauthorship network, highlighting key researchers and their collaborations within the field. (b) Cocitation network, identifying authors frequently cited together and their influence on the research landscape.

Figure 4 provides an in-depth analysis of author collaboration and cocitation patterns in the field of mitochondria and pain research from 2004 to 2024. Figure 4(a) illustrates the co-authorship network, highlighting key researchers who frequently collaborated within this field. Prominent authors included Mustafa Naziroglu, Annemieke Kavelaars, and Cobi J. Heijnen, who formed central nodes in the collaboration network. The overall network displayed a moderate degree of collaboration density, with several well-connected clusters centered around these core authors. However, a number of peripheral nodes with fewer links were also present, indicating some fragmentation and suggesting that certain research groups remain relatively isolated from the main collaborative hubs. Figure 4(b) depicts the cocitation network, identifying authors who were frequently cited together in the literature. Notable authors such as Flatters SJL, Zhang Y, and Bennett GJ were central nodes in this network, reflecting their influential contributions to the field.  Table 2 presents the top 10 authors and cocited authors on research of mitochondria and pain.

### 3.4. Journal Cocitation and Dual-Map Overlay Analysis in Mitochondria and Pain Research

**Figure 5:**
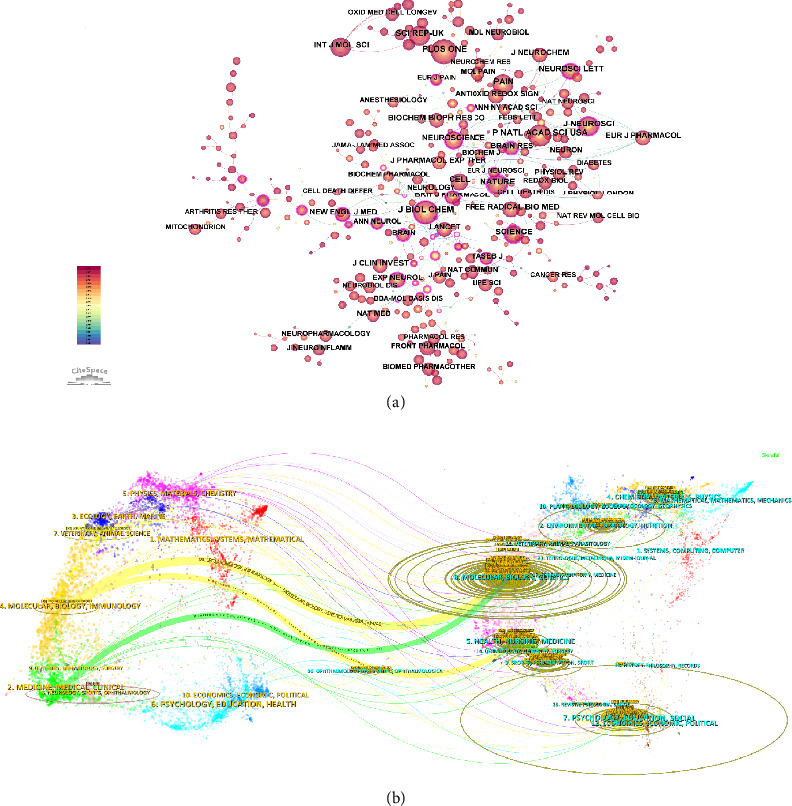
Journal cocitation and dual-map overlay analysis in mitochondria and pain research. (a) Journal cocitation network, indicating the most frequently cited journals and their connections. (b) Dual-map overlay, illustrating citation patterns between citing and cited journals, showing the flow of knowledge across different scientific fields.

Figure 5(a) reveals the journal cocitation network, highlighting the most frequently cited journals in this domain. Key journals such as The Journal of Biological Chemistry, Nature, and Science emerged as central nodes, reflecting their significant influence on the development of research in this area. The network structure underscored the interdisciplinary nature of mitochondria and pain research, with strong connections between journals focused on molecular biology, neuroscience, and clinical research. Figure 5(b) presents the dual-map overlay, which illustrated the citation patterns between citing journals on the left and cited journals on the right. The primary citation paths were observed from journals in fields such as molecular biology, genetics, and health, nursing, and medicine, indicating the flow of knowledge from basic scientific research to applied clinical studies. This overlay analysis emphasized the integrated approach in studying mitochondrial functions and their implications for pain, effectively bridging the gap between fundamental research and clinical applications. Collectively, these analyses highlighted the pivotal role of high-impact journals in shaping the research landscape of mitochondria and pain, revealing the interconnectedness of various scientific disciplines that contributed to this complex field.  Table 3 presents the top 10 journals and cocited journals on research of mitochondria and pain.

### 3.5. Cocitation and Clustering Analysis of Mitochondria and Pain Research Literature

**Figure 6:**
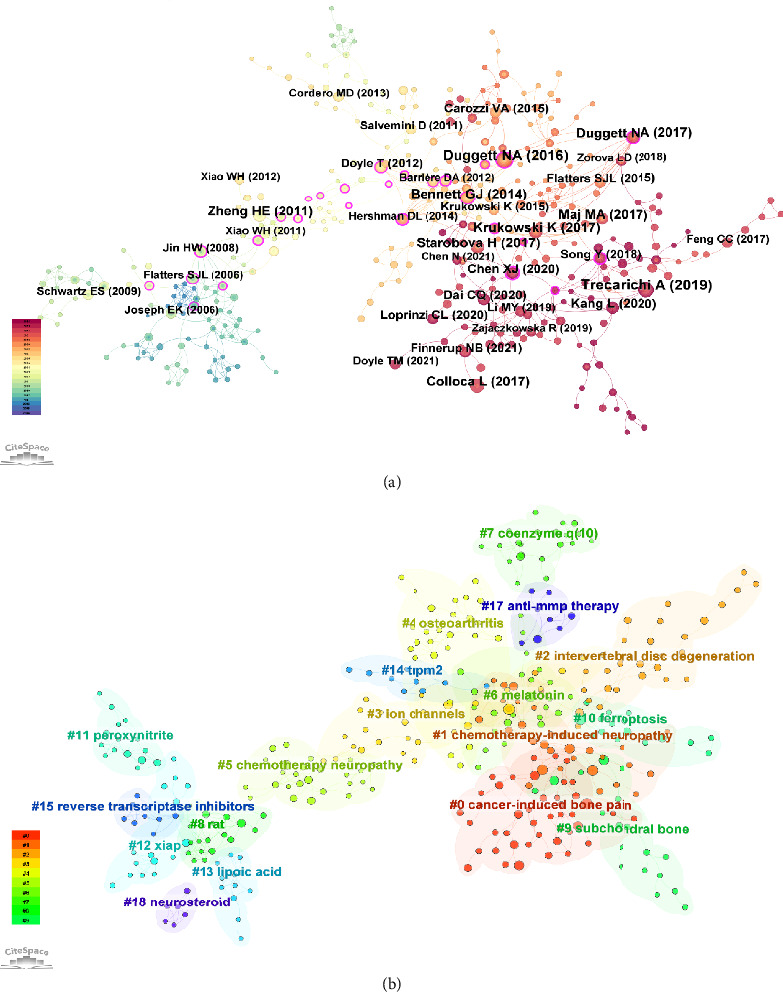
Co-citation and clustering analysis of mitochondria and pain research literature. (a) Co-citation network of the most frequently cited references, highlighting pivotal studies in the field. (b) Clustering of cocited references, revealing distinct thematic clusters within the research literature.

Figure 6(a) displays the cocitation network of the most frequently cited references within this domain. Key references, such as Trecarichi A (2019) in International Review of Neurobiology, Duggett NA (2016) in Neuroscience, and Bennett GJ (2014) in Nature Reviews Neurology, emerged as pivotal works frequently cited together. These studies served as foundational references in the field, significantly impacting subsequent research and guiding the exploration of the relationship between mitochondrial dysfunction and pain mechanisms. Figure 6(b) presents the clustering of these cocited references, revealing distinct thematic clusters within the research literature. For example, one prominent cluster included studies focusing on cancer-induced bone pain, while another cluster concentrated on the chemotherapy-induced neuropathy. These clusters highlighted the key thematic areas that have driven research in the field, illustrating how the literature has evolved over time and pointing to emerging research frontiers.

**Figure 7:**
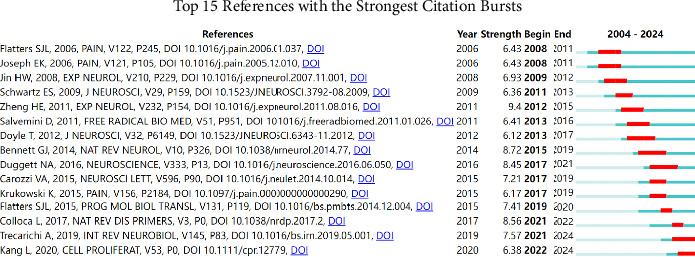
References with the strongest citation bursts in mitochondria and pain research. This figure identifies key studies that experienced significant increases in citations over time, reflecting their impact on the field.

Figure 7 highlights the references with the strongest citation bursts in mitochondria and pain research from 2004 to 2024. These bursts indicate periods during which certain references received an exceptionally high number of citations, reflecting their significant impact on the field. The analysis identified several key studies that experienced strong citation bursts, underscoring their influence. For instance, Bennett GJ (2014) in Nature Reviews Neurology demonstrated a notable citation burst from 2015 to 2019, marking it as a pivotal work in the understanding of mitochondrial mechanisms in neuropathic pain. Another prominent example is Hershman DL (2014) in the Journal of Clinical Oncology, which showed a strong citation burst from 2017 to 2019, reflecting its impact on research exploring the role of mitochondrial dysfunction in cancer-related pain. These citation bursts highlight the evolving focus of research within the field, with certain studies catalyzing new directions and methodologies. The temporal patterns of these bursts also provide insights into the research trends and the growing recognition of the importance of mitochondrial pathways in pain mechanisms.  Table 4 presents the top 10 cocited references on research of mitochondria and pain.

### 3.6. Keyword Analysis

**Figure 8:**
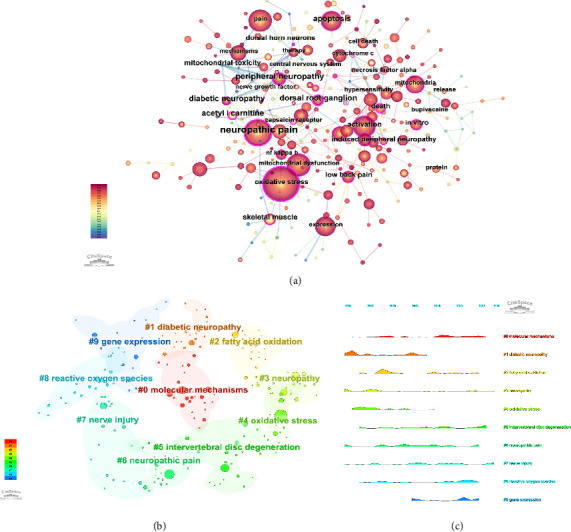
Keyword co-occurrence, clustering, and temporal analysis in mitochondria and pain research. (a) Keyword co-occurrence network, showing the most frequently appearing terms in the literature. (b) Keyword clustering, identifying distinct thematic groups within the research. (c) Temporal mountain plot of keyword clusters, illustrating the evolution of research themes over time.

Figure 8(a) illustrates the keyword co-occurrence network, revealing the most frequently appearing terms in the literature. Keywords such as “mitochondrial dysfunction,” “oxidative stress,” and “neuropathic pain” were central to the network, indicating their importance in the research field. Figure 8(b) presents the clustering of these co-occurring keywords, identifying distinct thematic groups within the research. The keyword clustering analysis yielded a modularity *Q* value of 0.45 and a mean silhouette score of 0.82, indicating that the clusters had a clear structural separation and high internal consistency. For instance, one cluster focused on molecular pathways involving mitochondrial dysfunction, while another concentrated on the clinical aspects of neuropathic pain. These clusters provided insights into the major research themes and how they have evolved over time, reflecting the interdisciplinary approach to studying mitochondrial involvement in pain. Figure 8(c) shows the same keyword clusters as in Figure 8(b), but displayed them in a temporal context using a mountain plot, illustrating how these research themes have evolved over time. The temporal analysis provided in Figure 8(c) highlighted the periods during which specific clusters gained prominence, showing the dynamic shifts in research focus.

**Figure 9:**
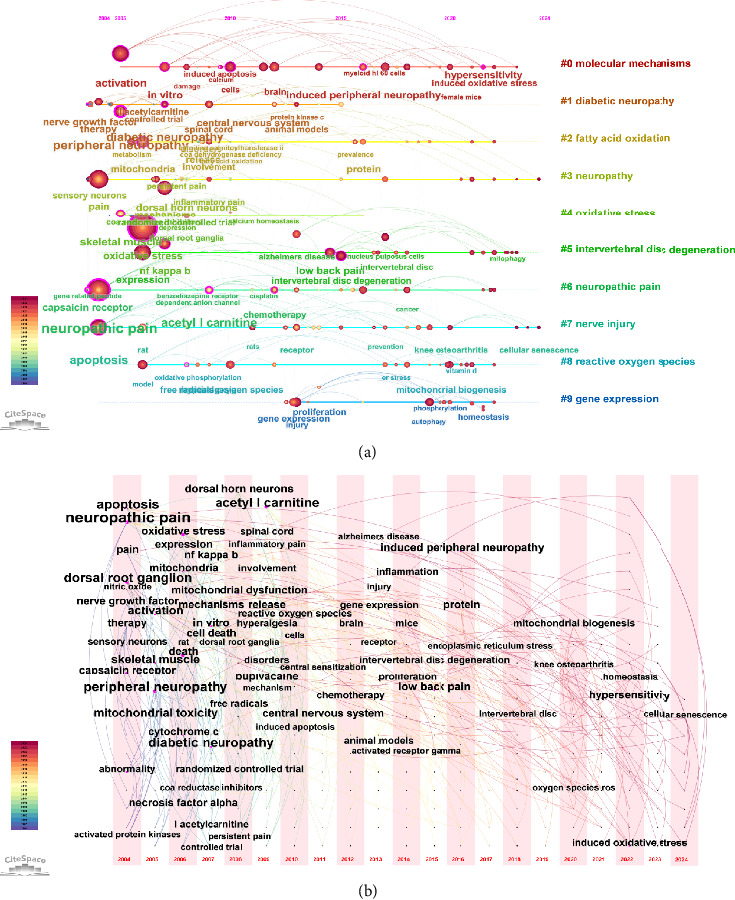
Temporal analysis of keywords in mitochondria and pain research. (a) Keyword timeline, showing how specific keywords have emerged and developed over time. (b) Keyword time-zone map, displaying the temporal distribution of research themes across different periods.

Figure 9(a) illustrates the keyword timeline, showing how specific keywords have emerged and developed over time. This timeline analysis revealed that early research focused on fundamental concepts such as “mitochondrial dysfunction” and “oxidative stress,” which have persisted as central themes throughout the years. As research progressed, newer keywords like “autophagy,” “neuroinflammation,” and “mitochondrial biogenesis” began to appear, reflecting the evolving understanding of mitochondrial roles in pain mechanisms. Figure 9(b) presents the time-zone map of keywords, which displayed the temporal distribution of research themes across different periods. This time-zone analysis highlighted the shifts in research focus, with earlier studies concentrating on basic mitochondrial functions and later studies expanding into more specific areas.

**Figure 10:**
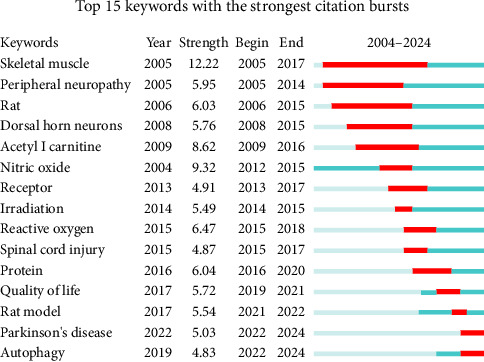
Keyword burst analysis in mitochondria and pain research. This figure highlights the periods during which specific keywords experienced significant increases in research attention, indicating emerging or intensifying trends within the field.

Figure 10 presents the keyword burst analysis, highlighting the periods during which specific keywords experienced significant increases in research attention within the field of mitochondria and pain. The analysis identified several keywords that exhibited strong bursts, indicating emerging or intensifying research trends. Notably, terms like “autophagy,” “Parkinson's disease,” and “rat model” showed pronounced bursts, particularly in recent years. These bursts reflect the growing interest and recognition of these areas as critical components in understanding the role of mitochondria in pain mechanisms.

## 4. Discussion

### 4.1. Overview of Bibliometric Findings

This bibliometric analysis provides a detailed examination of the research landscape surrounding mitochondria and pain from 2004 to 2024, revealing several key trends and patterns that have emerged over the past two decades. The study identified a clear upward trajectory in the number of publications, particularly from 2011 onwards, indicating a growing recognition of the importance of mitochondrial dysfunction in pain mechanisms. The surge in research activity between 2015 and 2020 highlights this area as a rapidly expanding field, driven by advances in mitochondrial science and an increasing interest in novel pain management strategies.

The analysis of international and institutional collaborations underscored the global nature of this research domain, with significant contributions from the United States, China, and several European nations. These countries have established themselves as central players in the field, frequently engaging in collaborative research efforts that have propelled the understanding of mitochondrial roles in pain. Notably, institutions such as Harvard University, the University of California, and the University of Texas were identified as key contributors, reflecting their leadership in fostering research that spans across various disciplines and geographic boundaries.

Author collaboration and cocitation analyses further highlighted the influence of specific researchers and foundational studies. Authors like Mustafa Naziroglu, Annemieke Kavelaars, and Cobi J. Heijnen were identified as central figures within the collaboration networks, indicating their significant roles in shaping the research agenda. Similarly, cocitation networks revealed the impact of pivotal works, such as those by Bennett GJ and Hershman DL, which have been extensively cited and have served as cornerstones for subsequent research.

Journal cocitation and dual-map overlay analyses revealed the interdisciplinary nature of the field, with strong connections between journals focused on molecular biology, neuroscience, and clinical research. High-impact journals such as The Journal of Biological Chemistry, Nature, and Science were frequently cited, reflecting their influence in disseminating critical findings and shaping the research directions in mitochondria and pain.

Finally, the keyword analysis provided insights into the thematic evolution of the field. Keywords such as “mitochondrial dysfunction,” “oxidative stress,” and “neuropathic pain” emerged as central themes, with more recent studies increasingly focusing on specialized topics like “autophagy” and “neuroinflammation.” The temporal analysis of keywords illustrated how research priorities have shifted over time, with emerging themes gaining prominence in response to new scientific discoveries and clinical needs.

### 4.2. Current Research Trends and Developments

The bibliometric analysis of mitochondria and pain research from 2004 to 2024 highlights several key trends and developments that have shaped the current research landscape. A significant trend identified in the analysis is the increasing focus on the molecular and cellular mechanisms underlying mitochondrial dysfunction and its role in various types of pain, particularly neuropathic and chronic pain.

One of the most prominent themes emerging from the analysis is the role of oxidative stress and mitochondrial dysfunction in pain mechanisms. This theme is consistently reflected in the keyword co-occurrence and clustering analyses, where terms like “mitochondrial dysfunction,” “oxidative stress,” and “neuroinflammation” are central to the research field. These findings indicate a growing recognition of the importance of mitochondria in maintaining cellular homeostasis and how their dysfunction can contribute to pain pathophysiology. Researchers are increasingly focusing on understanding the complex interactions between mitochondrial health, ROS production, and their effects on pain perception and nociceptor sensitization.

Mitochondrial dysfunction has emerged as a central theme in pain research, particularly in the context of neuropathic and chronic pain. This dysfunction is characterized by impaired ATP production, increased production of ROS, and disrupted mitochondrial dynamics, all of which contribute to the pathophysiology of pain [18, 19]. Recent studies have focused on the role of mitochondrial dysfunction in sensitizing nociceptors, the neurons responsible for pain perception. For example, mitochondrial dysfunction in dorsal root ganglion neurons has been linked to increased pain sensitivity in models of diabetic neuropathy [20, 21]. Researchers have also identified specific mitochondrial pathways, such as the MPTP and its regulation, as potential therapeutic targets for mitigating pain [22]. The use of MPTP inhibitors like cyclosporine A has shown promise in preclinical studies for reducing neuropathic pain [23]. Oxidative stress, closely linked to mitochondrial dysfunction, is another critical area of research. Mitochondria are the primary source of ROS within cells, and excessive ROS production can lead to oxidative damage of cellular components, contributing to chronic pain conditions [24]. Studies have demonstrated that oxidative stress plays a significant role in the sensitization of pain pathways, particularly in conditions like inflammatory and neuropathic pain. For instance, in models of chemotherapy-induced peripheral neuropathy, oxidative stress has been shown to exacerbate neuronal damage and pain [25]. Current research is exploring antioxidants as potential therapeutic agents to counteract oxidative stress. Compounds such as N-acetylcysteine (NAC) and mitoquinone (MitoQ) are being investigated for their ability to mitigate oxidative damage and alleviate pain symptoms [26, 27]. Neuroinflammation is increasingly recognized as a critical contributor to chronic pain, with mitochondria playing a central role in this process. Mitochondrial dysfunction can activate proinflammatory pathways, leading to the release of cytokines and chemokines that promote neuroinflammation [28]. This inflammation can, in turn, exacerbate pain by sensitizing nociceptors and disrupting normal neuronal function. Recent research has focused on the interplay between mitochondrial dysfunction and neuroinflammation in conditions such as multiple sclerosis and rheumatoid arthritis, where chronic pain is a major symptom [29, 30]. The identification of mitochondrial DNA as a damage-associated molecular pattern (DAMP) that can trigger innate immune responses has provided new insights into how mitochondrial dysfunction can perpetuate neuroinflammatory processes [31]. Targeting these pathways with anti-inflammatory agents or mitochondrial protectants is an emerging therapeutic strategy.

Another notable trend is the shift towards more specialized research topics, as evidenced by the emergence of keywords such as “autophagy,” “mitochondrial biogenesis,” and “calcium signaling” in more recent publications. These topics represent cutting-edge areas of investigation where scientists are delving deeper into the specific molecular pathways through which mitochondria influence pain. For example, the role of autophagy in mitochondrial quality control and its implications for chronic pain has become a critical area of study, as researchers seek to understand how dysregulated autophagy may contribute to sustained pain states [32, 33]. Autophagy, the process by which cells degrade and recycle damaged cellular components, including mitochondria (a process known as mitophagy), is a key area of investigation in the context of pain [34–36]. Dysregulation of autophagy has been implicated in the persistence of chronic pain, as it can lead to the accumulation of damaged mitochondria, thereby exacerbating oxidative stress and inflammation [37, 38]. Research has shown that enhancing autophagy can improve mitochondrial function and reduce pain in models of neuropathic pain. For example, the use of rapamycin, an autophagy inducer, has been demonstrated to attenuate pain in preclinical studies by promoting the clearance of damaged mitochondria [39]. Ongoing research is exploring the therapeutic potential of modulating autophagy to restore cellular homeostasis and alleviate pain. Mitochondrial biogenesis, the process by which new mitochondria are formed within cells, is another critical area of focus. Enhancing mitochondrial biogenesis has the potential to restore mitochondrial function and energy balance in cells affected by chronic pain [40, 41]. Research has identified several key regulators of mitochondrial biogenesis, including PGC-1*α* (peroxisome proliferator-activated receptor-gamma coactivator 1-alpha), which plays a crucial role in coordinating the expression of genes involved in mitochondrial replication and function. Studies have shown that upregulating PGC-1*α* can improve mitochondrial function and reduce pain in animal models. For example, exercise-induced activation of PGC-1α has been found to increase mitochondrial biogenesis and reduce pain sensitivity in neuropathic pain models [41]. This line of research holds promise for developing interventions that target mitochondrial biogenesis as a means of alleviating chronic pain.

The analysis also revealed a strong focus on the clinical implications of mitochondrial dysfunction, particularly in conditions like cancer-induced pain and chemotherapy-induced neuropathy. The clustering of cocited references and the identification of key studies with significant citation bursts highlight the ongoing efforts to translate basic mitochondrial research into clinical applications. Researchers are increasingly exploring mitochondrial-targeted therapies, such as antioxidants and agents that modulate mitochondrial permeability, as potential treatments for pain, particularly in difficult-to-manage cases of neuropathic and inflammatory pain.

Beyond these well-established themes, the analysis also points to emerging research directions that are gaining attention within the field. For example, the exploration of mitochondrial dynamics, including mitochondrial fission and fusion processes, is an area of growing interest. Disruptions in these processes have been linked to pain, and research is beginning to investigate how modulating mitochondrial dynamics can influence pain outcomes. Additionally, there is an increasing focus on the role of mitochondria in non-neuronal cells, such as glial cells, which are known to contribute to pain signaling and maintenance.

### 4.3. Strengths and Limitations

This bibliometric analysis of mitochondria and pain research from 2004 to 2024 offers several strengths that contribute to a comprehensive understanding of the field. This study integrates multiple bibliometric approaches (cocitation analysis, keyword clustering, temporal burst detection, and dual-map overlays) to generate a comprehensive and multidimensional profile of research trends in the field. Unlike previous reviews, our analysis spans a 20-year period and systematically tracks how key topics such as mitochondrial biogenesis and oxidative stress have evolved over time within the pain research domain. The analysis also benefited from the inclusion of a large dataset sourced from the Web of Science Core Collection, ensuring that the findings are based on high-quality, peer-reviewed research. Another strength of this study is its ability to highlight both established and emerging areas of research within the field of mitochondria and pain. By examining cocitation networks, keyword co-occurrence, and clustering patterns, this analysis provided insights into the dynamic shifts in research focus over time. The identification of strong citation bursts and the temporal evolution of key themes allowed for a nuanced understanding of how research priorities have changed, reflecting both advancements in scientific knowledge and evolving clinical needs. Moreover, the study's emphasis on international and institutional collaborations provided valuable insights into the global nature of research in this domain. The identification of leading countries and institutions, as well as their collaborative networks, underscored the importance of interdisciplinary and cross-border cooperation in advancing the field. This global perspective is particularly important in understanding how different regions contribute to the development of knowledge and how collaborative efforts can drive innovation.

However, this study also has several limitations that should be acknowledged. First, the analysis was limited to publications indexed in the Web of Science Core Collection, which, while comprehensive, may not capture all relevant studies, particularly those published in non-English languages or in journals not indexed by this database. This limitation could result in a potential bias towards certain regions or disciplines, underrepresenting the contributions from other parts of the world or emerging areas of research. Second, bibliometric analysis, by its nature, focuses on quantitative metrics such as citation counts and co-occurrence frequencies. While these metrics provide valuable insights into research trends and influence, they do not capture the qualitative aspects of research, such as the methodological rigor, the clinical relevance of findings, or the real-world impact of the studies. Consequently, while this analysis identifies influential publications and authors, it does not assess the quality or effectiveness of the research itself. Additionally, the use of citation data as a proxy for research impact has inherent limitations. Citation practices can vary widely between disciplines, and highly cited papers are not always synonymous with high-quality or impactful research. Moreover, citation counts can be influenced by various factors, such as the visibility of the publication or the prominence of the authors, rather than the intrinsic value of the research. Finally, while the analysis provides a snapshot of the research landscape from 2004 to 2024, it does not account for ongoing or very recent developments that have not yet been fully reflected in the citation data. As a result, some emerging trends or influential works that are just beginning to gain attention may not be captured in this analysis.

In conclusion, while this bibliometric study offers a robust overview of the research landscape in mitochondria and pain, its findings should be interpreted with consideration of these limitations. Future studies could expand on this work by incorporating a broader range of data sources, qualitative assessments of research impact, and more recent publications to provide an even more comprehensive understanding of this rapidly evolving field.

## 5. Conclusions

This bibliometric analysis offers a clear overview of the research trends in mitochondria and pain from 2004 to 2024. The study highlights the growing recognition of mitochondrial dysfunction as a critical factor in pain mechanisms, with increasing research focused on oxidative stress, neuroinflammation, autophagy, and mitochondrial biogenesis. The findings underscore the dynamic nature of the field, with a rise in publications and a shift toward specialized topics. Despite significant progress, challenges remain, particularly in translating basic research into clinical practice and expanding global collaborations. Based on our findings, future studies should focus on elucidating the precise molecular mechanisms linking mitochondrial dysfunction to different pain phenotypes, and on translating these mechanistic insights into targeted interventions that can be evaluated in clinical settings.

## Figures and Tables

**Table 1:** Top 10 countries and institutions on research of mitochondria and pain.

Rank	Country	Count (%)	Institution	Count (%)
1	United States	541 (27.1)	University of California (United States)	62 (3.8)
2	China	510 (25.5)	University of Texas (United States)	48 (3.3)
3	Italy	137 (6.8)	Huazhong University of Science & Technology (China)	38 (2.7)
4	Germany	102 (5.1)	Harvard University (United States)	35 (2.7)
5	United Kingdom	98 (4.9)	Shanghai Jiao Tong University (China)	28 (2.5)
6	Canada	83 (4.2)	University of London (United Kingdom)	26 (2.3)
7	Japan	82 (4.1)	Harvard Medical School (United States)	23 (2.2)
8	Spain	78 (3.9)	U.S. Department of Veterans Affairs (United States)	23 (1.5)
9	India	76 (3.8)	Institut National de la Sante et de la Recherche Medicale (Inserm) (France)	21 (1.4)
10	Brazil	71 (3.5)	Veterans Health Administration (VHA) (United States)	19 (1.3)

**Table 2:** Top 10 authors and cocited authors on research of mitochondria and pain.

Rank	Authors	Count	Cocited authors	Citations
1	Naziroglu Mustafa	23	Sarah JL Flatters	213
2	Annemieke Kavelaars	15	Mario D Cordero	187
3	Zengwu Shao	14	Wenhua Xiao	169
4	Cobi J Heijnen	13	Naziroglu Mustafa	168
5	Michael R Hamblin	13	Elizabeth K Joseph	149
6	Min Xie	12	Yingying Zhang	133
7	Jon D Levine	12	Yayun Wang	123
8	Mario D Cordero	11	Guido Cavaletti	119
9	Shuanglin Hao	11	Rurong Ji	116
10	Daniela Salvemini	11	Gary J Bennett	113

**Table 3:** Top 10 journals and cocited journals on research of mitochondria and pain.

Rank	Journal	Count (%)	IF	JCR	Cocited journal	Cocitation	IF	JCR
1	International Journal of Molecular Sciences	43 (2.2)	4.9	Q1	Pain	2878	3.7	Q1
2	Pain	39 (1.9)	5.9	Q1	Journal of Biological Chemistry	2103	8.0	Q1
3	Scientific Reports	28 (1.4)	3.8	Q1	Journal of Neuroscience	2070	3.6	Q1
4	Plos One	27 (1.3)	2.9	Q1	PNAS	1984	5.1	Q1
5	Molecular Neurobiology	25 (1.2)	4.6	Q1	Plos One	1770	64.8	Q1
6	Frontiers in Pharmacology	25 (1.2)	4.4	Q1	Nature	1487	11.1	Q1
7	Molecular Pain	24 (1.2)	2.8	Q2	International Journal of Molecular Sciences	1120	2.9	Q2
8	Journal of Ethnopharmacology	22 (1.1)	4.8	Q1	Science	1091	15.5	Q1
9	Antioxidants	18 (0.9)	6.0	Q1	Neuroscience	1080	5.9	Q1
10	Frontiers in Physiology	18 (0.9)	3.2	Q2	Scientific Reports	984	4.6	Q2

**Table 4:** Top 10 cocited references on research of mitochondria and pain.

Rank	Cocited reference	Citations
1	Chaplan SR, 1994, J Neurosci Meth, v53, p55	97
2	Flatters SJL, 2006, Pain, v122, p245	86
3	Zheng He, 2011, Exp Neurol, v232, p154	61
4	Zimmermann M, 1983, Pain, v16, p109	51
5	Kim HK, 2004, Pain, v111, p116	50
6	Xiao WH, 2012, Pain, v153, p704	48
7	Joseph EK, 2006, Pain, v121, p105	47
8	Flatters SJL, 2015, Prog Mol Biol Transl, v131, p119	46
9	Bennett GJ, 1988, Pain, v33, p87	46
10	Caterina MJ, 1997, Nature, v389, p816	43

## Data Availability

The datasets generated and analyzed during the current study are available from the corresponding author upon reasonable request. The bibliometric data were sourced from the Web of Science Core Collection, which is accessible through institutional subscriptions. All data supporting the findings of this study are included in the article.
